# Eustachian and Aortic Valve Endocarditis: A Rare Case of Concomitant Right- and Left-Sided Endocarditis

**DOI:** 10.7759/cureus.111310

**Published:** 2026-06-22

**Authors:** Nicolas Riveros Neira, Benjamin J Behers, Omar Hozayen, Karen Hamad, Natan Kraitman, Wilhelmine Wiese-Rometsch

**Affiliations:** 1 Internal Medicine, Florida State University Internal Medicine Residency/Sarasota Memorial Hospital, Sarasota, USA; 2 Infectious Diseases, Sarasota Memorial Hospital, Sarasota, USA

**Keywords:** bilateral infective endocarditis, eustachian valve vegetation, multivalvular endocarditis, streptococcus mutans, systemic embolization

## Abstract

Concomitant right- and left-sided infective endocarditis (IE) is an uncommon clinical entity, particularly when involving the Eustachian valve (EV), a vestigial remnant of the inferior vena cava. While right-sided IE is typically associated with intravenous drug use and indwelling cardiac devices, it can occur in non-traditional populations if anatomical niches are colonized. A 78-year-old female presented with heart failure and a "mixed" embolic signature, including pulmonary, renal, and cerebral infarcts, following a dental extraction. Imaging confirmed aortic valve endocarditis and a 1.5 cm vegetation on the EV. Blood cultures were positive for *Streptococcus mutans*. Despite lacking traditional risk factors for right-sided infection, the patient required emergent surgical intervention for refractory heart failure and high embolic burden. This case highlights the unique virulence of *S. mutans* and the necessity of comprehensive echocardiographic evaluation of fetal remnants when multisystem emboli are present.

## Introduction

Infective endocarditis (IE) remains a significant cause of morbidity, with an escalating incidence of 13.8 per 100,000 person-years [1]. This increase is largely driven by an aging demographic and the increased use of intracardiac hardware [1]. Typically, 90-95% of cases involve the left-sided valves (aortic or mitral) [1]. Right-sided IE is rare and often associated with intravenous drug use (IVDU), indwelling catheters, and implanted cardiac devices, usually targeting the tricuspid valve [1].

Multivalvular involvement occurs in only 10-18% of cases [1]. Aortic-tricuspid IE, although rare (1.5%), had the worst prognosis, with the highest rates of septic shock and mortality [1]. Relying on traditional risk profiles, such as the absence of IVDU, can lead to the underdiagnosis of right-sided involvement in complex presentations [1]. Eustachian valve (EV) endocarditis has traditionally been considered a rare form of right-sided IE [2]. Owing to its infrequent occurrence, data regarding its epidemiology and clinical characteristics remain limited, particularly in cases occurring concomitantly with left-sided IE [1,2]. Its low prevalence may reflect both the EV's status as an embryologic remnant that typically undergoes postnatal regression and the absence of routine, systematic echocardiographic assessment of the EV during evaluations for IE [2]. We present a rare case of concomitant aortic and EV endocarditis in a patient whose only identifiable risk factor was a recent dental procedure.

## Case presentation

A 78-year-old female with no reported past medical history presented with a three-week progression of dyspnea, palpitations, and lower extremity edema. Symptoms manifested shortly after a dental extraction for acute odontogenic pain. The patient reported that she did not receive antibiotic prophylaxis because she denied a history of cardiac disease. On admission, she was afebrile (98.8ºF), tachycardic (HR 115/minute), hypotensive (BP 96/62 mmHg), without acute respiratory distress, with an oxygen saturation of 97% on room air. Physical examination revealed confusion, diffuse pulmonary crackles, a grade 3/6 systolic ejection murmur, and 3+ pitting edema. No focal neurologic deficits or cutaneous abnormalities were identified.

Laboratory studies were significant for leukocytosis with neutrophilia, elevated proBNP, elevated D-dimer, and elevated CRP (Table 1). Blood cultures drawn on admission grew pansensitive *Streptococcus mutans*, a common oral commensal.

**Table 1:** Admission laboratories

Lab	Value	Normal Range
Leukocytes	17.1 x 10³ cells/µL	4.5–11.0 x 10^3^ cells/µL
Neutrophils	15.03 x 10^3^ cells/μL	1.50–7.0 x 10^3^ cells/µL
proBNP	22,598 pg/mL	0–1800 pg/mL
D-dimer	1.88 mg/L	0.00–0.49 mg/L
CRP	8.0 mg/dL	<0.3 mg/dL

The clinical course was defined by a bi-ventricular embolic shower. Thoracic computed tomography (CT) revealed a right middle lobe pulmonary embolus and interstitial edema (Figure 1). Abdominal CT revealed a possible left renal cortical infarct; renal function tests were within normal limits, and urinalysis was unremarkable without proteinuria or hematuria. Notably, the patient's initial presentation of confusion acutely worsened into a more profound altered mental status, prompting a brain MRI (Figure 2). Imaging revealed a punctate acute infarct in the medial right frontal lobe, providing a direct clinical-radiographic correlation for her acute cognitive and executive decline.

**Figure 1:**
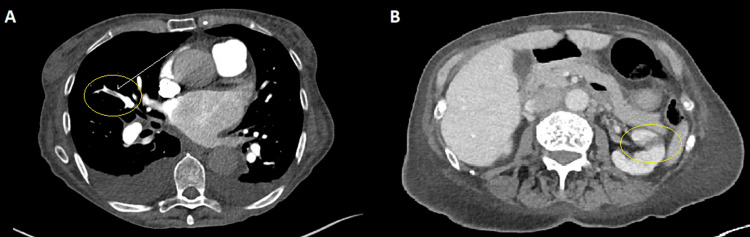
CTA thorax (A) CTA thorax showed a right middle lobe pulmonary artery embolus (yellow circle), moderate bilateral pleural effusions, interstitial edema, and cardiomegaly consistent with congestive heart failure. (B) CT abdomen and pelvis with IV contrast showed a left renal cortical wedge-shaped defect (yellow circle) to be correlated with possible infarct versus pyelonephritis.

**Figure 2:**
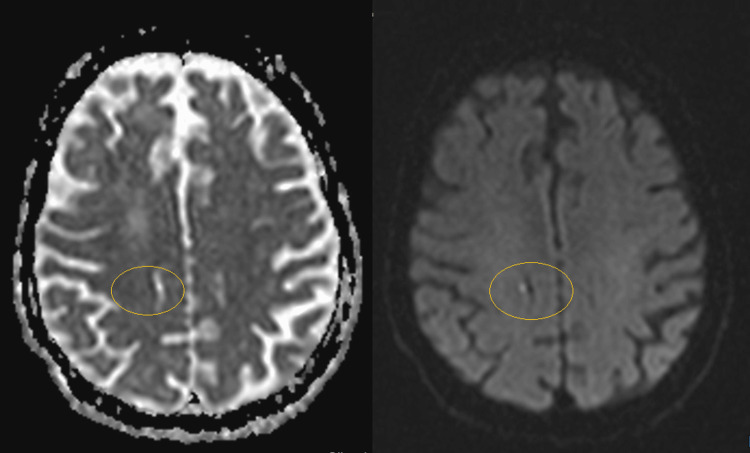
MRI brain without contrast Punctate focus of restricted diffusion in the posterior medial right frontal lobe, suggestive of an acute infarct (yellow circle).

Transthoracic and transesophageal echocardiography demonstrated a left ventricular ejection fraction of 25-30%, a mildly dilated right ventricle with mildly depressed function, a moderately enlarged left atrium, a severely enlarged right atrium, and an intact atrial septum without a PFO. Additionally, it showed moderate to severe mitral regurgitation, severe aortic stenosis with multiple vegetations, and a 1.5 cm x 0.9 cm mass on the EV (Figure 3). These findings were confirmed on a transesophageal echocardiogram (Figure 4). Based on the echocardiographic findings, it was considered to have possibly had previously undiagnosed aortic stenosis.

**Figure 3:**
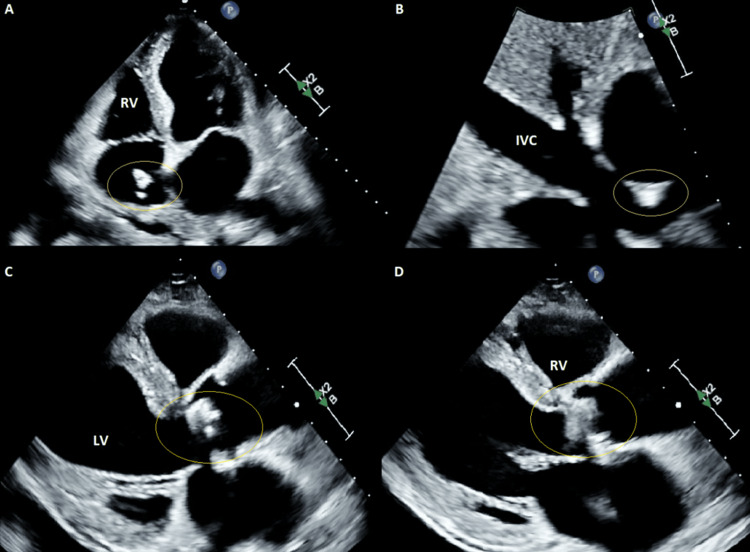
Trans-thoracic echocardiogram (A) A 1.5 cm × 0.9 cm mass was noted on the Eustachian valve (yellow circle). (B) The Eustachian valve and the anatomical relation with the IVC (yellow circle). (C) Severe aortic valve stenosis with multiple vegetations on the aortic valve with partial outlet obstruction (yellow circle). D. Multiple vegetations on the aortic valve (yellow circle). IVC: inferior vena cava; RV: right ventricle; LV: left ventricle

**Figure 4:**
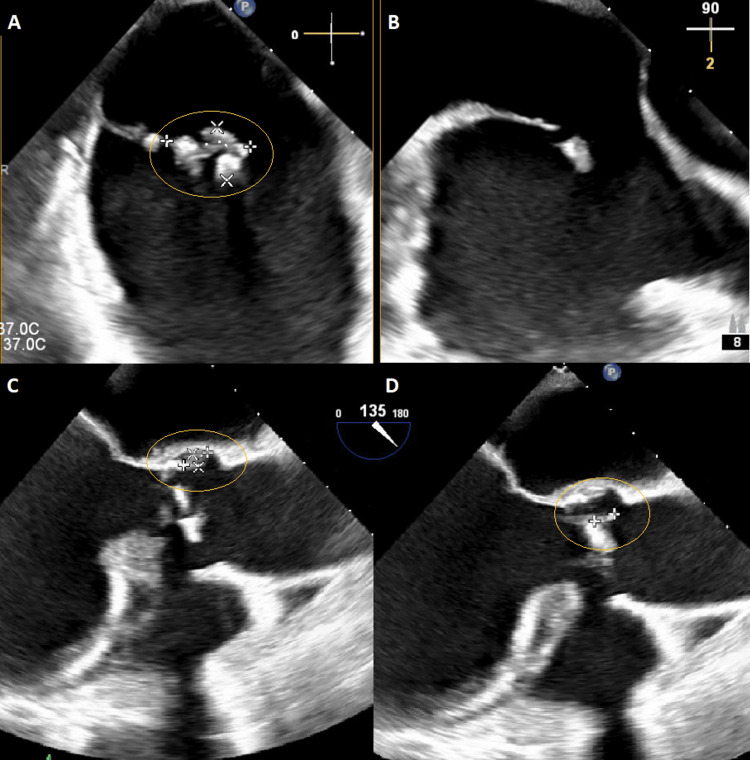
Trans-esophageal echocardiogram (A) Prominent eustachian valve with the appearance of a large 1.5 × 0.9 cm (yellow circle), very mobile mass attached at its terminus. (B) Eustachian valve. (C) 0.8 x 0.4 cm aortic valve mass (yellow circle). (D) 0.6 x 0.2 cm aortic valve mass (yellow circle).

The infectious diseases team was consulted, and she was started on empiric cefepime, vancomycin, and doxycycline. Once blood cultures were available, the patient was transitioned to ceftriaxone 2 g IV daily. Cardiology was also consulted and assisted with diuresis and up-titration of guideline-directed medical therapy for this newly diagnosed cardiomyopathy. Due to refractory heart failure and the high risk posed by the large EV vegetation, cardiothoracic surgery was consulted, and the patient underwent aortic valve replacement, mitral valve repair, and excision of the Eustachian mass. Notably, during surgery, the EV mass was identified, and it was noted that the endocardium was extensively inflamed with multiple soft adhesions and an area of possible abscess in the left coronary sinus.

Following surgical intervention and clearance of bacteremia, as confirmed by repeat blood cultures on hospital day 4, the patient's mentation progressively improved. EV mass and aortic valve leaflet pathology showed valve tissue with calcification, fibrosis, and acute suppurative inflammation. She returned to her neurologic baseline without residual focal deficits and was ultimately discharged after 24 days to complete an extended course of intravenous antibiotics and to follow up with infectious diseases, cardiology, and cardiothoracic surgery.

## Discussion

This scenario highlights an important limitation of established algorithms, necessitating a high-fidelity diagnostic approach. The EV is a fetal remnant located where the inferior vena cava enters the right atrium [2]. EV endocarditis is exceedingly rare, accounting for approximately 3.3% of right-sided cases, though its true incidence may be underestimated as the structure is frequently overlooked without routine transesophageal echocardiography [2,3]. In this patient, the absence of IVDU or indwelling cardiac devices, the typical drivers of right-sided infections, initially focused clinical attention on the aortic valve, highlighting a potential pitfall in the differential diagnosis process [2,4].

The pathogen profile identified in this case, *Streptococcus mutans*, serves as a critical diagnostic "pearl." While *S. mutans* is a common oral commensal associated with dental procedures, it harbors a unique clinical signature [3,5]. Despite causing less than 8% of streptococcal bacteremias, it poses an endocarditis progression risk, estimated as high as 47% [6]. Historically, it affects left-sided valves in patients with underlying valve disease or damaged endothelium, especially following recent dental procedures [7,8]. However, colonization of the EV, as seen in our case, is exceedingly rare and scarcely reported in the existing literature [2,9]. Clinical implementation of this knowledge requires a low threshold for comprehensive imaging when this specific organism is isolated, even after seemingly routine odontogenic events [7,8].

Furthermore, the quality of care in complex endocarditis is fundamentally linked to the early recognition of a "mixed" embolic signature. Simultaneous presentation of pulmonary emboli and systemic infarcts, such as the renal and cerebral events, may indicate possible multivalvular involvement or the presence of a patent foramen ovale [10-12]. This bi-ventricular embolic shower traces distinct pathophysiological pathways: the right frontal and renal infarcts were downstream consequences of the left-sided vegetations, while the pulmonary emboli directly showered from the EV mass. Implementing a comprehensive diagnostic mandate is essential in these scenarios to fully capture and characterize this divergent embolic burden [13].

The surgical strategy and subsequent follow-up in this case were guided by the established IDSA/AHA (Infectious Diseases Society of America/American Heart Association) 2015 guidelines, which included the presence of refractory heart failure due to valvular dysfunction and a vegetation size exceeding 10 mm [13]. The patient's successful outcome underscores the relevance of early, multidisciplinary cardiovascular team involvement. Following surgical intervention, care quality must extend into the longitudinal setting, focusing on structural integrity via serial imaging, antimicrobial stewardship to prevent relapse of this virulent pathogen, and the up-titration of guideline-directed medical therapy for the associated cardiomyopathy. Ultimately, this case demonstrates that anatomical niches can serve as a nidus for infection regardless of traditional risk profiles, and a high index of suspicion for vestigial structures is required to optimize diagnostic accuracy and patient outcomes.

## Conclusions

We highlight a rare case of simultaneous right- and left-sided endocarditis of the Eustachian and aortic valves following a recent tooth extraction. Systemic and pulmonary emboli following IE diagnosis emphasize the need for a broad differential and a comprehensive echocardiographic evaluation, including transesophageal imaging when suspicion remains high, to identify uncommon anatomical niches such as the EV, even in the absence of IVDU or device-related risk factors. Our case shows the importance of a multidisciplinary team approach to endocarditis, especially in cases with multiple complications. Following valve replacement and targeted antimicrobial therapy, the patient recovered successfully and was discharged with ongoing cardiology and infectious diseases follow-up. This case underscores the need for heightened awareness of vestigial cardiac structures as potential sites of infection and reinforces the value of a comprehensive diagnostic approach in patients presenting with mixed embolic phenomena.

## References

[1] Dobreva-Yatseva B, Nikolov F, Raycheva R, Tokmakova M (2024). Infective endocarditis—characteristics and prognosis according to the affected valves. Microorganisms.

[2] San Román JA, Vilacosta I, Sarriá C, Garcimartín I, Rollán MJ, Fernández-Avilés F (2001). Eustachian valve endocarditis: is it worth searching for?. Am Heart J.

[3] Sawhney N, Palakodeti V, Raisinghani A, Rickman LS, DeMaria AN, Blanchard DG (2001). Eustachian valve endocarditis: a case series and analysis of the literature. J Am Soc Echocardiogr.

[4] Venkatram M, Kommuri NV, Kollepara SL (2009). Eustachian valve endocarditis: a rare complication of automatic implantable cardioverter defibrillator placement. J Heart Valve Dis.

[5] Schmidt MA, Nigbor D, Eitzman DT (2001). Eustachian valve endocarditis caused by Streptococcus viridans. J Am Soc Echocardiogr.

[6] Deas G, Lee TC, Colston J (2025). Streptococcal endocarditis: a meta-analysis of species dependant risk. EClinicalMedicine.

[7] Duval X, Millot S, Chirouze C (2017). Oral streptococcal endocarditis, oral hygiene habits, and recent dental procedures: a case-control study. Clin Infect Dis.

[8] Baltimore RS, Gewitz M, Baddour LM (2015). Infective endocarditis in childhood: 2015 update: a scientific statement from the American Heart Association. Circulation.

[9] Kim SL, Gordon SM, Shrestha NK (2018). Distribution of streptococcal groups causing infective endocarditis: a descriptive study. Diagn Microbiol Infect Dis.

[10] Li M, Kim JB, Sastry BK, Chen M (2024). Infective endocarditis. Lancet.

[11] Habib G, Erba PA, Iung B (2019). Clinical presentation, aetiology and outcome of infective endocarditis. Results of the ESC-EORP EURO-ENDO (European infective endocarditis) registry: a prospective cohort study. Eur Heart J.

[12] Bohbot Y, Habib G, Laroche C (2022). Characteristics, management, and outcomes of patients with left-sided infective endocarditis complicated by heart failure: a substudy of the ESC-EORP EURO-ENDO (European infective endocarditis) registry. Eur J Heart Fail.

[13] Baddour LM, Wilson WR, Bayer AS (2015). Infective endocarditis in adults: diagnosis, antimicrobial therapy, and management of complications: a scientific statement for healthcare professionals from the American Heart Association, endorsed by the Infectious Diseases Society of America. Circulation.

